# Development of balanced nutrient management innovations in South Asia: Perspectives from Bangladesh, India, Nepal, and Sri Lanka

**DOI:** 10.1016/j.gfs.2020.100464

**Published:** 2021-03

**Authors:** Avinash Kishore, Muzna Alvi, Timothy J. Krupnik

**Affiliations:** a International Food Policy Research Institute (IFPRI), NAS Complex, New Delhi, 110008, India; b International Maize and Wheat Improvement Center (CIMMYT), Sustainable Intensification Program, Dhaka, 1213, Bangladesh

**Keywords:** Fertilizer subsidy, Soil health cards, Balanced nutrient management, Fertilizer policy, Organic fertilizers, Bio-fertilizers

## Abstract

Imbalanced application of fertilizers is a major fiscal and environmental problem in South Asia. We review fertilizer policies and extension efforts to promote the balanced application of nutrients in Bangladesh, India, Nepal, and Sri Lanka and draw 4 important lessons. (1) Fertilizer sector reforms need to be fiscally sustainable and politically feasible. Governments in South Asia have abolished fertilizer subsidies on multiple occasions, only to restore them a few years later. (2) The use of phosphate and potash did not decline much even after a sharp increase in their prices in India in 2011–12. Therefore, rationalizing subsidies, while necessary, may not be sufficient to ensure balanced use of fertilizers. Changing farmers' practice requires combining the right incentives with the right information. (3) Soil test based soil health cards (SHC) hold promise, but there is limited evidence on their utility. India's SHC program had very little impact on fertilizer use. (4) Direct cash transfer (DCT) of fertilizer subsidies can reduce distortions, but Sri Lanka's experience shows that implementing it is more challenging than universal subsidies. DCT requires the removal of price controls, integration of land records, farmer identity cards, a cash transfer system with universal coverage, and a competitive fertilizer retail sector.

## Highlights

• Imbalanced application of fertilizers is a major problem across South Asia.
• Farmers do not have the scientific information on plant nutrient requirements.
• High fertilizer subsidies distort farmers incentives.
• Implementing direct cash transfer of fertilizer subsidies requires a good database of farmers and universal access to institutional finance.
• Farmers adopt fertilizer use recommendations only if the information is presented in a simple format through a series of interactions with them.

## 1 Introduction

Imbalanced application of fertilizers is a common problem across South Asia. Subsidy induced distortions of the incentives of the farmers and the fertilizer industry and poor information on soil nutrient requirements are two of the main reasons for this problem that hurts the farmers, the taxpayers, and the local and the global environment. Over the last few years, Bangladesh, India, Nepal, and Sri Lanka have tried new, large-scale, and often costly revisions of fertilizer policies and started ambitious extension programs to improve farmers’ access to fertilizers and promote balanced application of soil nutrients (Gulati and Banerjee, 2016; Huang et al., 2017; Kyle et al., 2017; Kanthilanka, and Weerahewa, 2019). This paper reviews some of the major policy changes and extension efforts and synthesizes evidence from stakeholder engagements and a series of national and regional dialogues with policymakers, extension organizations, private fertilizer companies, and farmers on soil fertility and the use of balanced nutrient management in South Asia.

## 2 Key lessons on nutrient management and fertilizer use in cereal-based cropping systems in South Asia

### 2.1 High macronutrient subsidies are common across South Asia

Bangladesh, India, Nepal, and Sri Lanka heavily subsidize nitrogen, phosphate, and potash fertilizers. The highest subsidies are in Sri Lanka (78–83 percent of world prices) where the three main fertilizers (urea, DAP/TSP, and MoP) are sold at the same controlled price of 5 cents/kg. Prices are nearly four times higher in Bangladesh (18–19 cents/kg) and urea is only marginally cheaper than TSP and MoP. On the other hand, urea is significantly cheaper than DAP and MoP in India and Nepal (Table 1).

**Table 1:** Prices ($/kg) of urea, DAP/TSP, and MOP in Bangladesh, India, Nepal, and Sri Lanka in 2020.

Fertilizer type	Price (US Dollar kg^−1^)			
	Bangladesh	India	Nepal	Sri Lanka
Urea	0.18	0.07	0.11	0.05
DAP/TSP	0.19	0.29	0.35	0.05
MOP	0.19	0.21	0.25	0.05

Note: For Sri Lanka, prices are for paddy farmers only, for all other crops, prices are USD 0.15 per kg. The mentioned prices are TSP prices for Sri Lanka and DAP for all other countries. Live market exchange rates as of April 25, 2020, used for conversion.

In the past, fertilizer subsidies helped promote the use of fertilizers and contributed to significant increases in yields (Morris et al., 2007), but in recent years, their contribution to productivity increase (Kapur, 2011; Sharma, 2012) and overall agricultural growth and poverty reduction has declined over time (Fan et al., 2007) even as the subsidy bill keeps growing. Apart from exacting high economic costs, the existing subsidy regimes in Bangladesh, India, Nepal, and Sri Lanka also incentivize excessive application of urea and underapplication of P and K fertilizers (at least in India and Nepal), micronutrients, and organic inputs; contribute to soil degradation, contamination of groundwater, and emission of greenhouse gases (Chand and Pandey, 2009; Prasad, 2009; Liu et al., 2014); discourage product innovation by fertilizer companies; and crowd out productive investments in agricultural research and development (Gulati and Banerjee, 2016).

There is also a large body of evidence showing that benefits from fertilizer subsidies tend to accrue to certain kinds of farmers (Sharma and Thaker, 2010; Praveen et al., 2017), crops (Pingali, 2012), and regions (Takeshima et al., 2017), and to fertilizer manufacturers more than farmers (Gulati and Narayanan, 2000). Therefore, a central question for subsidy reforms is how to allocate limited budgets more efficiently for better and more effective targeting. In a region where fertilizer policy, and until recently, fertilizer production was state-controlled, there is also the question of what role can the private sector play in promoting balanced nutrient application.

A major constraint to the restructuring of input subsidies has been a lack of understanding of how farmers make decisions about which and how much fertilizers to use and how they respond to price (Duflo et al., 2011) and non-price signals. For example, we know from previous research in other contexts that the impact of subsidies can be strengthened when complemented with information (Ashraf et al., 2013; Stuart et al., 2014). There is however very little research on how incentives and information interact in the South Asian context (Nasrin et al., 2019; Pandey and Diwan, 2020).

### 2.2 Abolition of fertilizer subsidies tends to be temporary

Despite all the problems, the abolition of fertilizer subsidies is unlikely in the South Asian countries. Over the last 30 years, Bangladesh, Nepal, and Sri Lanka have all experimented with the abolition of fertilizer subsidies, only to restore them a few years later due to economic or political considerations (Huang et al., 2017; Mujeri et al., 2013). Bangladesh abolished fertilizer subsidies in 1992 and reinstated them in 2001 (Manos et al., 2007; Alam, 2018). Similarly, Nepal did not have fertilizer subsidies between 1997–98 and 2008–09 (Kyle et al., 2017). Sri Lanka had no fertilizer subsidies from 1990 to 1994 and subsidies only for urea from 1997 to 2005 (Weerahewa et al., 2010). India has never abolished fertilizer subsidies since they started in 1977^1^ (Sharma and Thaker, 2009). However, in 2010, Government of India decontrolled P and K prices and allowed them to rise sharply with the world prices, but urea prices were left untouched because the decisionmakers “felt that decontrolling of entire urea sector is extremely sensitive and NBS in urea is not feasible” (page 9, Lok Sabha Secretariat 2020). Fertilizer policy reforms in India and its neighboring countries need to be not only fiscally and environmentally sustainable, but also politically feasible. Globally also, input subsidies have become, not just a tool to enhance productivity and improve access but also an instrument guided by considerations of political expediency (Islam, 2014; Mason, Jayne and van de Walle, 2013; Takeshima and Liverpool-Tasie, 2015; Tarrant, 1982).

High fiscal burden and pressure from lending institutions were some of the reasons why countries abolished fertilizer subsidies. Subsidies were also reduced or removed when world prices were low and had to be restored when prices started escalating (Kyle et al., 2017). Unfortunately, we do not have careful case studies of these episodes of the abolition and subsequent reinstatement of fertilizer subsidies and we know very little about their impact on farmers’ welfare, farming practices, or balanced use of nutrients. Addressing this knowledge gap could help in devising more practical and economically sound recommendations for future reforms.

### 2.3 Farmers have tepid responses to price changes

The government of India decontrolled prices of Phosphatic and Potassic (P and K) fertilizers in 2011, resulting in a sharp increase in the retail prices of both nutrients. Now, Government of India fixes the total subsidy allocations for phosphate (P) and potash (K) fertilizers and allows retail prices to float with world prices (Ministry of Chemicals and Fertilizers, Government of India, 2019).

**Fig. 1:**
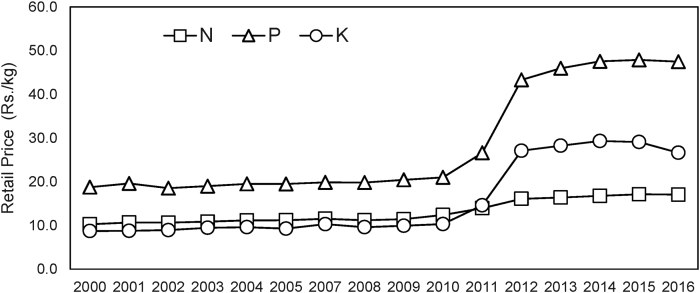
Limited observed responses to sharp changes in fertilizer prices in India: Prices of Urea, DAP, and MoP in India (June 2008–June 2017). Source: Commission for Agricultural Costs and Prices (CACP), several rounds. Note: DAP = Phosphate; MoP = Muriate of Potash; Rs = Indian Rupees.

**Fig. 2:**
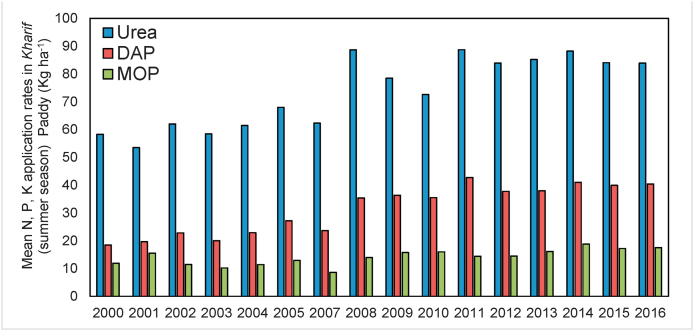
Average application rates (kg/ha) of N, P, and K in Kharif Paddy in India (2008–2017). Source: Commission for Agricultural Costs and Prices (CACP), several rounds.

Visual examination of field-level data on fertilizer use in India shows that the sharp increase in relative prices of P and K (Fig. 1) resulted in only small changes in the application rates of the two nutrients to rice (Fig. 2), the largest crop in India (FAOSTAT 2020). Farmers’ response to the significant changes in the relative prices of N, P, and K fertilizers^2^ in India appears to be surprisingly small.

This lack of response to major changes in relative prices of fertilizers suggests that rationalizing subsidies, though necessary, may not, by itself, lead to more balanced application of nutrients in India. Using multi-criteria modeling, Manos et al. (2007) also show that increasing fertilizer prices alone may not be the most appropriate policy strategy to change fertilizer consumption in Bangladesh because consumption does not change substantially until prices reach a level where they negatively affect farm income.

Lack of information and understanding of crop nutrient requirements and appropriate application timings among farmers is another reason for the suboptimal use of fertilizers (Emran et al., 2019; Jat et al., 2015; Mujeri et al., 2013). Besides incentives, farmers also need scientific information to change their fertilizer use and adopt agronomic practices that affect the nutrient recovery efficiency of cereal crops (Acheampong et al., 2017; Ladha et al., 2020; Yang and Fang, 2015).

### 2.4 Soil health information alone does not change farmers’ practices

All four countries in the region have public programs to disseminate soil wet chemistry test–based fertilizer use recommendations. In 2015, the Government of India launched a large-scale program where 23.6 million soil samples were tested and 93 million soil health cards (SHCs) test results and fertilizer recommendations were distributed to farmers in the first phase. Studies show that giving soil tests–based recommendations to farmers without adequate education on what test data mean or how to make use of them has a negligible effect on both farmers’ understanding of crop nutrient requirements and their actual use (Fishman et al., 2016).

**Fig. 3:**
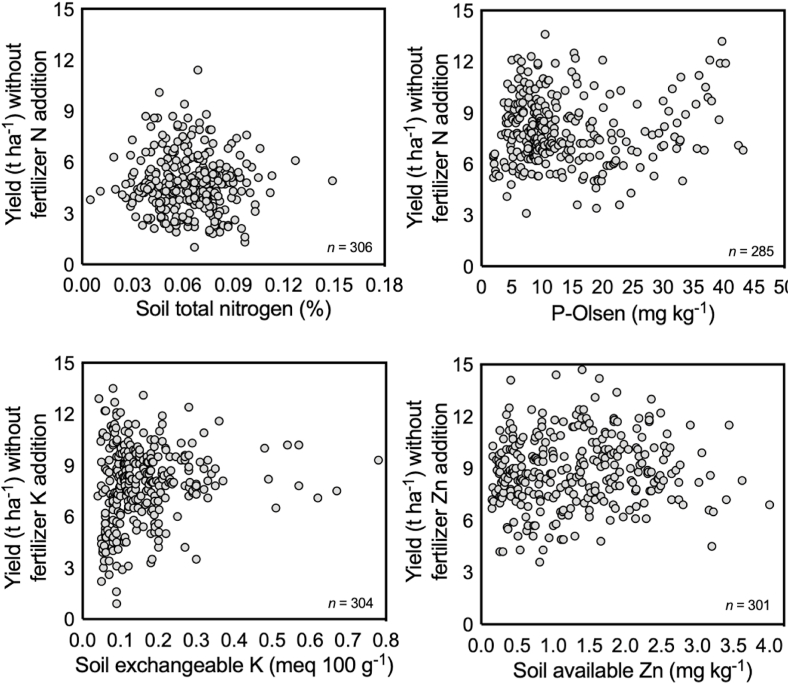
Yields achieved by irrigated maize farmers across Bangladesh during the 2011–12 and 2012–13 winter crop season Note: Recorded from nitrogen, phosphorous, potassium, and zinc nutrient omission plots (in which the full recommended dose for all non-omitted nutrients were applied). Total soil N, P-Olsen, soil exchangeable K and soil available Zn were measured by the Bangladesh Agricultural Research Institute following methods recommended by SRDI (2014).

Soil test data may also be an unreliable or weak predictor of crop yields. Work conducted by the Cereal Systems Initiative in South Asia (CSISA) in Bangladesh has found only a weak relationship between soil nutrient test results and yield of maize grown when N, P, K, and Zn fertilizers are sequentially not applied (Fig. 3). We would expect to see a stronger relationship between test results and yields if the soil test results were good indicators of yield without the addition of these nutrients. Without such a relationship, the development of appropriate soil fertility recommendations based on soil tests alone is compromised. Fig. 3 provides an example of the problems associated with commonly applied soil nutrients that are subsequently used to develop fertilizer recommendations, supporting well-established research (Doberman et al., 2003; Cassman et al., 1995). The lack of correlation seen in Fig. 3 can result from a mismatch between soil sampling time, depth, frequency, and the types of wet chemistry tests conducted (Briat et al., 2020; Schut and Giller, 2020; Reddy, 2017; CSISA, 2019a), in addition to the large influence of farmers’ crop management practices and climate on yield.

Agricultural development initiatives that focus on soil fertility in South Asia, however, still rely widely on soil test results, indicative of methodological and technological ‘lock-in’ (Barnes, 2004), with insufficient attention to alternatives (Shut and Giller, 2020). This does not necessarily imply that soil wet chemistry tests or the SHC concept are not useful; rather, where soil test information is considered, careful and conservative interpretation of the results of soil tests are needed, alongside efforts to increase farmers' understanding of other abiotic and biotic factors that affect yield and crop responsiveness to fertilizers. Research in Odisha and Gujarat states of India shows that simplifying SHCs and making them more user-friendly led to significant improvements in farmers' comprehension of soil health information (Singh et al., 2018; Cole and Sharma, 2017). Repeated engagement with farmers through call centers or personal visits by extension or fertilizer company staff have been shown to result in increases in understanding of SHCs and a small, but significant, increase in the adoption of more balanced nutrient application recommendations **(**Cole and Sharma, 2017**)**. Experience deploying app-based fertilizer recommendations to large numbers of farmers in different parts of India and Bangladesh also shows the need for a repeated engagement with farmers and field-level extension agents to increase understanding of soil fertility and create impact (Singh et al., 2018).

CSISA has started working on high-throughput soil nutrient stock assessment using reflectance spectroscopy and the development of high-resolution nutrient mapping using predictions based on geostatistical models and a statistical sample at the landscape level (Nanni and Demattê, 2006; Nocita et al., 2015). This approach aids in the generation of digital soil maps predicting soil fertility parameters – and also their uncertainties – even for areas where none or few physical samples have been taken and measured (CSISA, 2018). These pixel-based maps are high resolution, and when combined with time-series data, can be used to develop dynamic soil and crop management recommendations (Sanchez et al., 2009) This approach is, thus, likely to be useful in large densely populated countries such as Bangladesh or India where a) farmers’ soil management practices are highly variable; b) it is difficult to obtain plot-level samples from a representative sample of farmers, and c) chemical labs for soil testing are already overburdened.

### 2.5 Challenges in popularizing custom blends and micro-nutrient fortified fertilizers

Availability of location and crop-specific custom fertilizer blends and micronutrient fortified fertilizers at competitive prices can make it easier for the farmers to act on SHC recommendations. Customized fertilizers were introduced in India in 2008 and the government moved from a product pricing system to a nutrient based subsidy (NBS) regime in 2010 to promote innovation and increase the availability of new fertilizer products in the market at reasonable prices. Under the NBS, the government announces a fixed rate of subsidy (in Rs/kg) on each nutrient (N, P, K, and S), and the per kg subsidy rate is converted into the per ton subsidy on different fertilizer products.

The NBS policy also provides an additional subsidy of Rs. 300 ton^−1^ for boron and Rs. 500 ton^−1^ for zinc in fertilizers fortified or coated with zinc or boron. Companies can fortify 20% of their production and are allowed to sell these fertilizers at 10% above the notified maximum retail price (Lok Sabha Secretariat, 2020). Today, 36 grades of blended fertilizers and 23 fortified fertilizers are available in India, but even after 10 years, the total production and use of micronutrient fortified fertilizers remain low. There are three reasons for this: one, urea, the most commonly used fertilizer, is still under price control; two, the Rs. 300–500/ton of subsidy and the 10% price premium together do not cover the cost of coating the fertilizer; and three, the higher tax on micronutrient mixtures (18%) compared to straight micronutrient fertilizers (12%) disincentivizes the production and sale of fortified fertilizers (PWC, 2019).

The All India Coordinated Research Project on Micronutrients (AICRP-M) by the ICAR has shown widespread micronutrient deficiencies in India: 36.5% of the cultivated land is deficient in zinc, 24.2% of the land is deficient in Boron and 12.8% of the land is deficient in iron. ICAR research has also shown that need-based application of micronutrients will increase the efficiency of N, P, and K fertilizers, reduce the cost of cultivation, increase crop yield and crop quality and nutritional value. The ICRISAT has implemented a large project in the state of Karnataka in India to promote the application of zinc, boron, and sulphur in deficient areas and shown large yield gains to farmers (Wani et al., 2016). Government of Karnataka distributes the three micronutrients at a 50% subsidy through its own field outlets in deficient areas. Five other states of India—Andhra Pradesh, Haryana, Madhya Pradesh, Goa, and Tamil Nadu—also provide varying levels of subsidy on different secondary and micronutrients. However, the evidence on the impact of these subsidy programs is scant, and careful benefit-cost analyses are yet to be done.

Nepal also plans to set up its first fertilizer-blending plant, thus putting the issue of custom blends at the center-stage of the fertilizer policy debate in the region. However, the production and uptake of blended and micronutrient fortified fertilizers are likely to remain low in India, Nepal, and other countries in South Asia till urea and other macronutrients remain under price control. Price controls make blended fertilizers uncompetitive (CSISA, 2019). While one solution is to subsidize blended products, much like unblended fertilizers, the high and rising fiscal burden of existing macronutrient subsidies lowers the possibility that governments will offer more subsidies on blended or fortified fertilizers. Some states in India have started subsidizing micronutrients, but they control the procurement and distribution with little participation of private companies and often resort to rationing to remain within the budget constraint. Such a controlled and rationed delivery system does not encourage product innovation and private sector participation, and therefore, has only limited impact **(**Gupta et al., 2020).

Popularizing custom blends in South Asia requires rationalization of fertilizer subsidies and removal of price controls. It also requires the deployment of faster and more reliable soil testing technologies and new methods for developing location- and crop-specific fertilizer use recommendations. Digital soil maps can help create the knowledge base needed for production of customized fertilizer blends (Iticha and Takele, 2019).

### 2.6 Implementing direct cash transfer of fertilizer subsidies requires preparation

The Government of Nepal is exploring the use of vouchers for fertilizer subsidies, while the Government of India has initiated a move to direct cash transfer (DCT) of fertilizer subsidies in a phased manner. Sri Lanka experimented with DCT for two years (four crop seasons) but reverted to the previous system of price subsidies in early 2018.

From 2005 to 2015, paddy farmers in Sri Lanka were able to buy N, P, and K fertilizers at $3 per 50 kg bag. In February 2016, Sri Lanka switched to direct cash transfer for fertilizer subsidies. Retail prices of fertilizers increased 6.5-fold and farmers received a cash transfer directly to their bank accounts for up to 2 ha of cultivated land. Economists often prefer cash transfers to in-kind subsidies because the former is less price distorting (Cunha et al., 2017; Dehejia, 2011). However, in Sri Lanka, the government continued to control fertilizer prices even after switching to DCT. During this period, prices of all three macronutrients remained equal, but at a significantly higher level than before. Furthermore, while initially, only paddy growers were eligible for the cash transfer, later, the government added a few more crops to the eligibility list. These restrictions and targeting requirements created a heavy burden of data collection and monitoring for extension workers, resulting in irregularities and delays in subsidy delivery. The government of Sri Lanka returned to the old system of price subsidies soon after losing local body elections in 2018. Fertilizer prices in Sri Lanka are even lower now than they were before the introduction of cash transfers, with N, P, and K all sold at US$2.75 for a 50 kg bag.

Sri Lanka's example offers key lessons for other countries. First, a reliable database of farmers with their land records is needed to better target new subsidy regimes to benefit smallholders. Secondly, if vouchers are to be targeted to a select group of farmers, initial targeting criteria should be as simple as possible. The use of complicated indices for targeting could lead to more discretion for lower bureaucrats and more errors and potential for misuse (Niehaus et al., 2013). Thirdly, allowing fertilizer subsidies only for rice in the early phases of the program in Sri Lanka made subsidy distribution more cumbersome (cropping pattern data had to be collected every season) and led to increased irregularities.^3^ Thus, a crop-neutral voucher policy should be explored.

Unlike Sri Lanka, the Government of India plans to shift to cash transfers in phases using new technologies to reduce transaction costs and irregularities. The government has installed point of sales (POS) machines in all 0.22 million fertilizer retail outlets^4^ in the country. At present, farm input dealers must validate all fertilizer sales to farmers using a biometric-based unique identification document called *Aadhar* to control leakages and fraud. Process evaluations of the implementation of *Aadhar*-linked fertilizer sales have found that poor internet connectivity has been a major challenge in the full implementation of Aadhar-linked sales of fertilizers (Giri et al., undated; Singh and Ward, 2018). *Aadhar* cards are yet to be linked to land records and soil health cards in most states. As a result, targeting of fertilizer subsidies and using cash transfers to promote recommended fertilizer rates remains a challenge.

Although theoretically promising, DBT of targeted subsidies, therefore, appears to be more challenging than universal subsidies. This is further complicated if operational landholding is used as the criterion for targeting because land records are not digitized and integrated with other farmer identity cards or citizen cards in most of South Asia (Singh and Ward, 2018).

### 2.7 Interest in organic fertilizers, the inclusion of pulses, and improved organic matter management is growing

There is a growing interest in organic and regenerative farming practices in South Asia. In 2015, the Government of India launched a dedicated scheme called *Paramaparagat Krishi Vikas Yojana* (PKVY), or ‘traditional agricultural development scheme’,^5^ to develop models of organic farming aimed at improving soil fertility. GoI launched another scheme in 2016 to provide a subsidy of Rs. 1500 ton^−1^ of city compost to fertilizer manufacturing and marketing companies to scale up production and consumption of the product. 0.8 million tons of city compost had been sold to farmers under this scheme until October 2019 (Lok Sabha Secretariat, 2020). The state governments of Andhra Pradesh, Maharashtra, and Sikkim now have programs promoting organic farming.^6^ Independent organic farm entrepreneurs have also emerged in peri-urban areas bordering cities, catering to an increasingly health-conscious urban clientele (Ravi, 2017).

Regular application of organic inputs can improve soil structure and water holding capacity (Otinga et al., 2013; Brar et al., 2015; Lal, 2015). Organic amendments alone are, however, unlikely to be sufficient to maintain high levels of productivity in intensively managed field cropping systems (Connor, 2008). On a nutrient per nutrient basis, organic fertilizers are expensive compared to synthetic fertilizers (Rowlings et al., 2019), and their application is more labor-intensive particularly for field crops (Reddy et al., 2014; Zhang et al., 2014), though they also supply secondary and micro nutrients. Low or absent price support, high labor requirements, and high costs of organic fertilizers skew farmers’ preferences towards synthetic materials. For these reasons, integrated approaches that combine the use of organic inputs and synthetic fertilizers are warranted (Shennan et al., 2017).

Opportunities also exist for more tightly coupling the flow of nutrients within individual farms or villages through the appropriate use of farmyard manure and recycling of crop residues (Jat et al., 2014; Saha et al., 2018; Turmel et al., 2017). The benefits of nitrogen-fixing that can be accrued when pulses are included in crop rotations are also well known (Jat et al., 2014; Hossain et al., 2016), but purposeful use of legumes to improve soil fertility is limited in South Asia probably because of high production and price risks in pulse cultivation.

## 3 Implications

Changes in fertilizer policies in Bangladesh, India, Nepal, and Sri Lanka over the last three decades shows that despite all problems, elected governments in these countries are unlikely to abolish fertilizer subsidies. An *ex-ante* analysis in India (Sharma, 2012) shows that the removal of fertilizer subsidy will make farming unprofitable in many states and may hurt small and marginal farmers in the agriculturally less developed parts of India. Similarly, Manos et al. (2007) show that a sharp increase in fertilizer prices may hurt farmers’ profits and reduce employment in agriculture. If true, the dismantling of fertilizer subsidies may not be economically desirable or politically feasible. This leaves us with two options for rationalizing subsidies.

Option one is to allow a gradual increase in the price of urea over the next few years and transfer the money saved on subsidy to farmers through other channels to garner their support for this change. The Government of India has successfully implemented this strategy to reduce diesel subsidies. This approach may also work for Bangladesh and Sri Lanka. Nepal, however, cannot follow this strategy unilaterally unless fertilizer prices also start rising in India, given the widespread informal import of fertilizers from across the border. Twenty years ago, when Nepal abolished fertilizer subsidies and deregulated the fertilizer sector, it did not lead to the development of a domestic fertilizer industry. Rather, it led primairly to an increase in the influx of Indian fertilizers and a sharp decline in legal imports (Kyle et al., 2017). Fertilizer policy changes in Nepal may even be counterproductive for both farmers and the fertilizer industry if they are not aligned with policies and prices in India.

The second option is to switch to non-distortionary direct cash transfer of fertilizer subsidies. Sri Lanka tried this between 2015–16 and 2018–19 and Governments of India and Nepal are also keen on implementing a system of fertilizer vouchers or some other form of direct transfer. Sri Lanka's recent experience shows that the DCT of fertilizer subsidies needs to be coupled with decontrolled prices. Sri Lanka retained price controls even after implementing DCT, though this was largely unsuccessful. DCT with price controls, therefore, appears to be essentially an accounting exercise that does not change farmers' or industry's incentives.

Both Sri Lanka's experiment and India's ongoing efforts to create infrastructure and database for DCT of fertilizer subsidies in phases shows that implementation is a major challenge in South Asia where land records are not up to date, digitized, or linked to farmers' identity card. Land tenancy records may also be poor, fertilizer sales are not always recorded, and cash transfers to farmers are often delayed. Millions of farmers also remain unconnected to a reliable and efficient cash transfer system (Kishore et al., 2013). Furthermore, designing a successful DCT system requires decisions on the right amount (or range) of transfers, the targeting criteria, and the mechanism to deliver money to farmers given the limitations of data, financial access, and technology in rural South Asia. An important design question is how would farmers' fertilizer use change if market prices increase after DCT (price elasticity), and would this change affect crop production and productivity? The Standing Committee on Chemicals and Fertilizers in India's parliament raised this question and found that “*No study has been conducted regarding the consumption of fertilizers by the farmers vis-à-vis cost of fertilizers* (emphasis original: page 45, Lok Sabha Secretariat, 2020).

Another relevant issue for DCT in South Asia is its impact on informal tenants and sharecroppers. Would the cash transfer indirectly benefit them as well? If so, to what extent? Finding credible answers to these questions is critical to design an equitable DCT system that does not lead to a reduction in farmers' incomes or crop productivity. In India, removing price subsidies will also render half of the domestic urea producing units unviable because of their high costs of production and increase the country's dependence on imports which may even drive up the world prices.

Fertilizer policy reforms in South Asia could be supported by investment in creating a new soil intelligence system that combines high-resolution digital soil maps with efforts to develop customized recommendations and fertilizer blends. Maps can also aid in improving farmers’ returns by factoring in fertilizer costs and crop prices in the generation of recommendations. In India and potentially in Bangladesh, soil intelligence systems are likely to be feasible using relatively rich data soils resources available with national research programs.

Decontrolling fertilizer prices, high-resolution data on soil and crop nutrient requirements, and integrated soil fertility approaches could be combined with the development of customized fertilizer blends and micronutrient coated fertilizers, ultimately making it easier for farmers to act on scientific recommendations. The rapid development of new fertilizer blends will also require a faster and more credible regulatory system for the approval of new products (CSISA, 2019b).

## Declaration of competing interest

The authors declare that they have no known competing financial interests or personal relationships that could have appeared to influence the work reported in this paper.

## Footnotes

1 Subsidies for phosphatic and other complex fertilizers started in 1979 and Single Super Phosphate in 1982 (Sharma and Thaker, 2009).

2 Nitrogen is applied mostly as Urea. A small fraction of N also comes from diammonium phosphate (DAP) which has 18% N by weight. DAP is the main source of phosphate in India followed by triple super phosphate (TSP), and Potash is applied in the form of Muriate of Potash.

3 Based on Avinash's personal communication with agricultural department officials in Kandy, Sri Lanka in September 2018.

4 Open Government Data Platform India. 2019. “Details of Number of Dealers in Each District of India.” Accessed October 7, 2019. https://data.gov.in/catalog/details-number-dealers-each-district-india?filters%5Bfield_catalog_reference%5D=87050&format=json&offset=0&limit=6&sort%5Bcreated%5D=desc.

5 https://darpg.gov.in/sites/default/files/Paramparagat%20Krishi%20Vikas%20Yojana.pdf.

6 See, for example, Maharashtra state: Hindu Business Line. 2018. “Maharashtra Mission for Organic Farming.” August 21. https://www.thehindubusinessline.com/news/maharashtra-mission-for-organic-farming/article24746974.ece
